# Supplementary material for: Open-channel structure of a pentameric ligand-gated ion channel reveals a mechanism of leaflet-specific phospholipid modulation
Source: Nat Commun. 2022 Nov 17;13:7017. doi: 10.1038/s41467-022-34813-5 (PMC9668969; doi:10.1038/s41467-022-34813-5)
Supplement: Supplementary file 1 — Supplementary Information [file 41467_2022_34813_MOESM1_ESM.pdf]

Supplementary Information

Open-channel structure of a pentameric ligand-gated ion channel reveals a mechanism of leaflet-specific phospholipid modulation

John T. Petroff II[1], Noah M. Deitzen[1], Ezry Santiago-McRae[2], Brett Deng[1], Maya S. Washington[1], Lawrence J. Chen[1], K. Trent Moreland[1], Zengqin Deng[3,4], Michael Rau[5], James A. J. Fitzpatrick[3,5,6,7], Peng Yuan[3,4], Thomas T. Joseph[8], Jérôme Hénin[9], Grace Brannigan[2,10], Wayland W. L. Cheng[1*]

[1]Department of Anesthesiology, Washington University School of Medicine, Saint Louis, MO, USA
[2]Center for Computational and Integrative Biology, Rutgers University, Camden, NJ, USA.
[3]Department of Cell Biology and Physiology, Washington University School of Medicine, Saint Louis, MO, USA
[4]Center for the Investigation of Membrane Excitability Diseases, Washington University School of Medicine, Saint Louis, MO, USA
[5]Center for Cellular Imaging, Washington University School of Medicine, Saint Louis, MO, USA
[6]Department of Neuroscience, Washington University School of Medicine, Saint Louis, MO, USA
[7]Department of Biomedical Engineering, Washington University School of Medicine, Saint Louis, MO USA
[8]Department of Anesthesiology and Critical Care, Perelman School of Medicine, University of Pennsylvania, Philadelphia, Pennsylvania 19104, United States
[9]Laboratoire de Biochimie Théorique, Institut de Biologie Physico-Chimique, CNRS UMR 8251 and Université Paris Diderot, 5013 Paris, France
[10]Department of Physics, Rutgers University, Camden, NJ, USA

*To whom correspondence should be addressed: Professor Wayland W. L. Cheng, Department of Anesthesiology, Washington University School of Medicine, MSC 8054-0043-12, Saint Louis, MO 63110. Telephone: (314)273-7958; E-mail: wayland.cheng@wustl.edu

Supplementary Table 1: **Cryo-EM data collection and refinement statistics**

	WT CA POPC EMD-27216 PDB 8D64	WT Apo POPC EMD-27215 PDB 8D63	WT CA 2:1:1 EMD-27218 PDB 8D66	WT Apo 2:1:1 EMD-27217 PDB 8D65	ELIC3 CA 2:1:1 EMD-27219 PDB 8D67	ELIC5 CA 2:1:1 EMD-27220 PDB 8D68
**Data collection and processing**						
Magnification	105000	105000	105000	105000	105000	105000
Voltage (kV)	300	300	300	300	300	300
Electron exposure ($e^{-}/Å^2$)	66	66	66	66	66	66
Defocus range (μm)	-1 to -2.5	-1 to -2.5	-1 to -2.5	-1 to -2.5	-1 to -2.5	-1 to -2.5
Pixel size (Å)	1.1	1.1	1.1	1.1	1.1	1.16
Symmetry imposed	C5	C5	C5	C5	C5	C5
Initial particle images (no.)	1636317	2896094	607649	1388052	1749806	2149491
Final particle images (no.)	252748	482792	124191	165015	165001	195134
Map resolution (Å)	3.19	3.14	3.14	3.47	3.3	3.36
FSC threshold	0.143	0.143	0.143	0.143	0.143	0.143
**Refinement**						
Initial model used	PDB 2YN6	This study	This study	This study	This study	This study
Model resolution (Å)	3.2	3.14	3.2	3.5	3.3	3.3
FSC threshold	0.5	0.5	0.5	0.5	0.5	0.5
Map sharpening B factor ($Å^2$)	-206	-166	-125	-128	-162	-165
Model composition						
Non-hydrogen atoms	12545	12785	12800	12780	12770	13010
Protein residues	1535	1535	1535	1535	1555	1560
Ligands	5	5	10	5	5	10
B factors ($Å^2$) (0.5)						
Protein	50.65	59.91	55.46	50.66	48.92	34.80
Ligand	20	20	20	20	20	20
R.m.s. deviations						
Bond lengths (Å)	0.008	0.007	0.009	0.009	0.01	0.008
Bond angles (°)	1.136	1.16	1.16	1.18	1.22	1.16
**Validation**						
MolProbity Score	1.82	1.53	1.83	1.84	1.63	1.58
Clashscore	7.30	6.67	7.66	9.42	3.98	3.37
Poor rotamers (%)	0	0	0	0	0	0
Ramachandran plot						
Favored (%)	93.77	94.75	93.77	95.08	92.94	93.01
Allowed (%)	6.23	5.25	6.23	4.92	7.06	6.99
Disallowed (%)	0	0	0	0	0	0

Supplementary Fig. 1

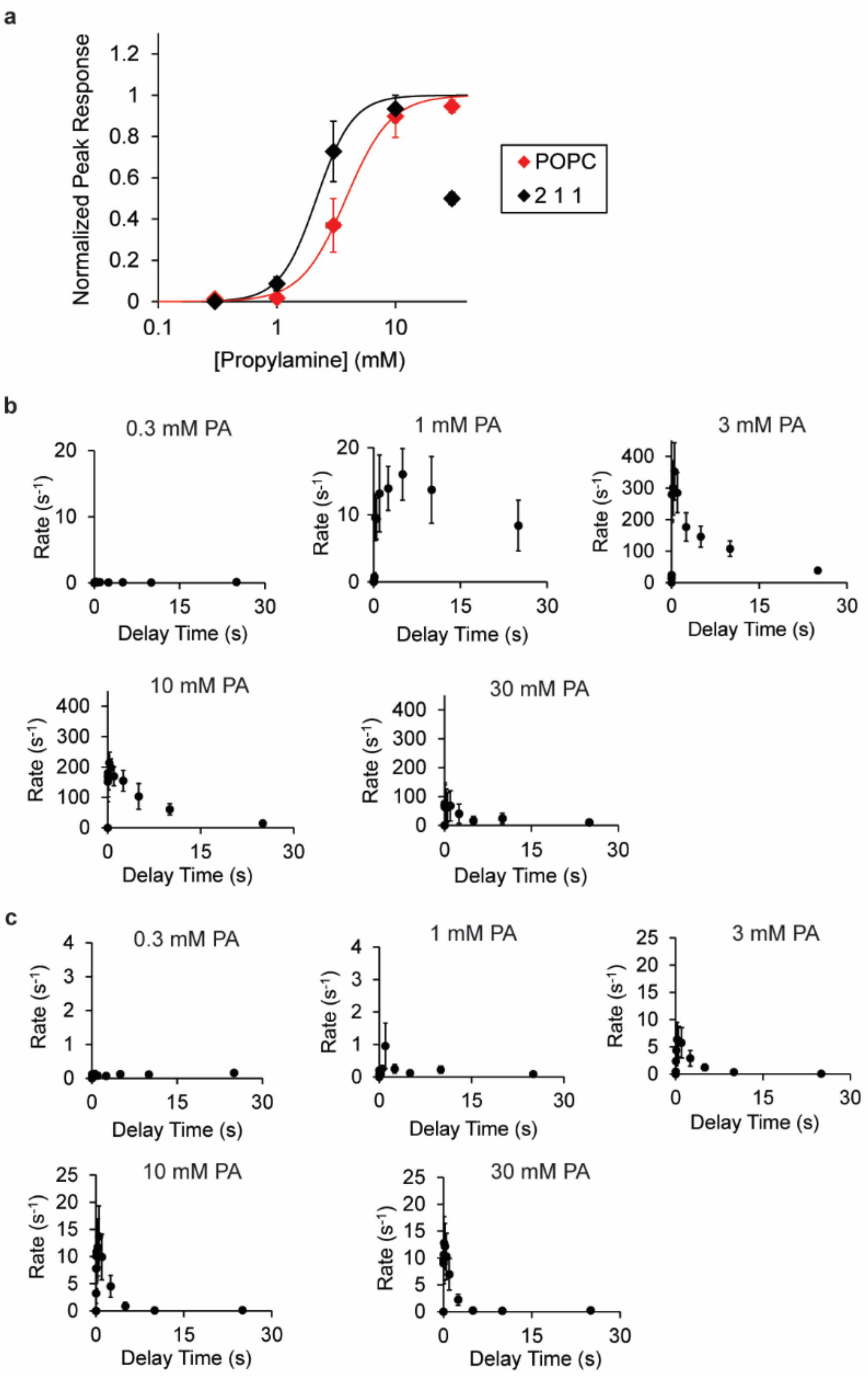

Supplementary Fig. 1 | Agonist sensitivity in 2:1:1 and POPC liposomes (a) Normalized plots of peak rates of ELIC in response to propylamine in 2:1:1 or POPC liposomes in the Tl^+ flux assay (n = 3). Data are fit to a Hill equation yielding an EC_{50} of 2.4 ± 0.4 mM and n of 2.2 ± 0.7 for 2:1:1, and an EC_{50} of 4.3 ± 1.4 mM and n of 3.6 ± 0.6 for POPC (p=0.2, unpaired T-test). **(b)** Tl^+ flux rates of WT ELIC in 2:1:1 POPC:POPE:POPG liposomes as a function of delay time after mixing with varying concentrations of propylamine (PA) (n = 3). **(c)** Tl^+ flux rates of WT ELIC in POPC liposomes as a function of delay time after mixing with varying concentrations of propylamine (n = 3). Data are shown as mean ± s.e. for (n) independent experiments. Source data are provided as a source data file.

Supplementary Fig. 2

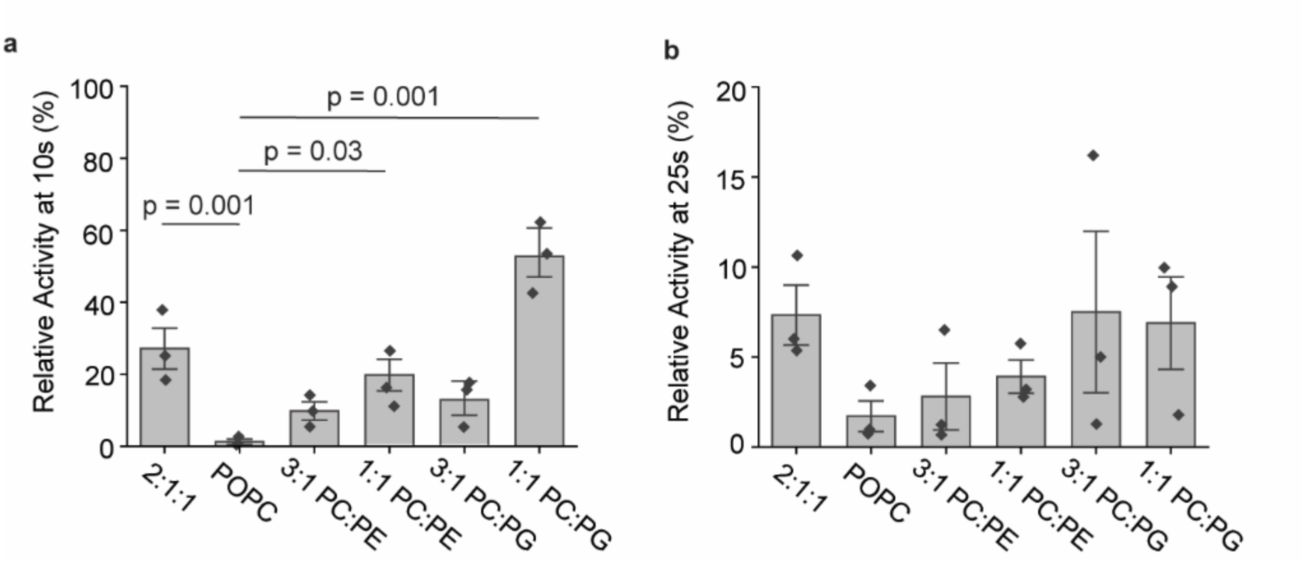

Supplementary Fig. 2 | ELIC activity in different lipid conditions (a) Relative activity at 10 s from Tl^+ flux data in Fig. 1c, measured as the rate at 10 s divided by the peak rate (n = 3). **(b)** Relative activity at 25 s from Tl^+ flux data in Fig. 1c, measured as the rate at 25 s divided by the peak rate (n = 3). Data are shown as mean ± s.e. for (n) independent experiments. Statistical analysis was performed using a one-way ANOVA and post-hoc Tukey test. P-values below 0.05 are shown. Source data are provided as a source data file.

Supplementary Fig. 3

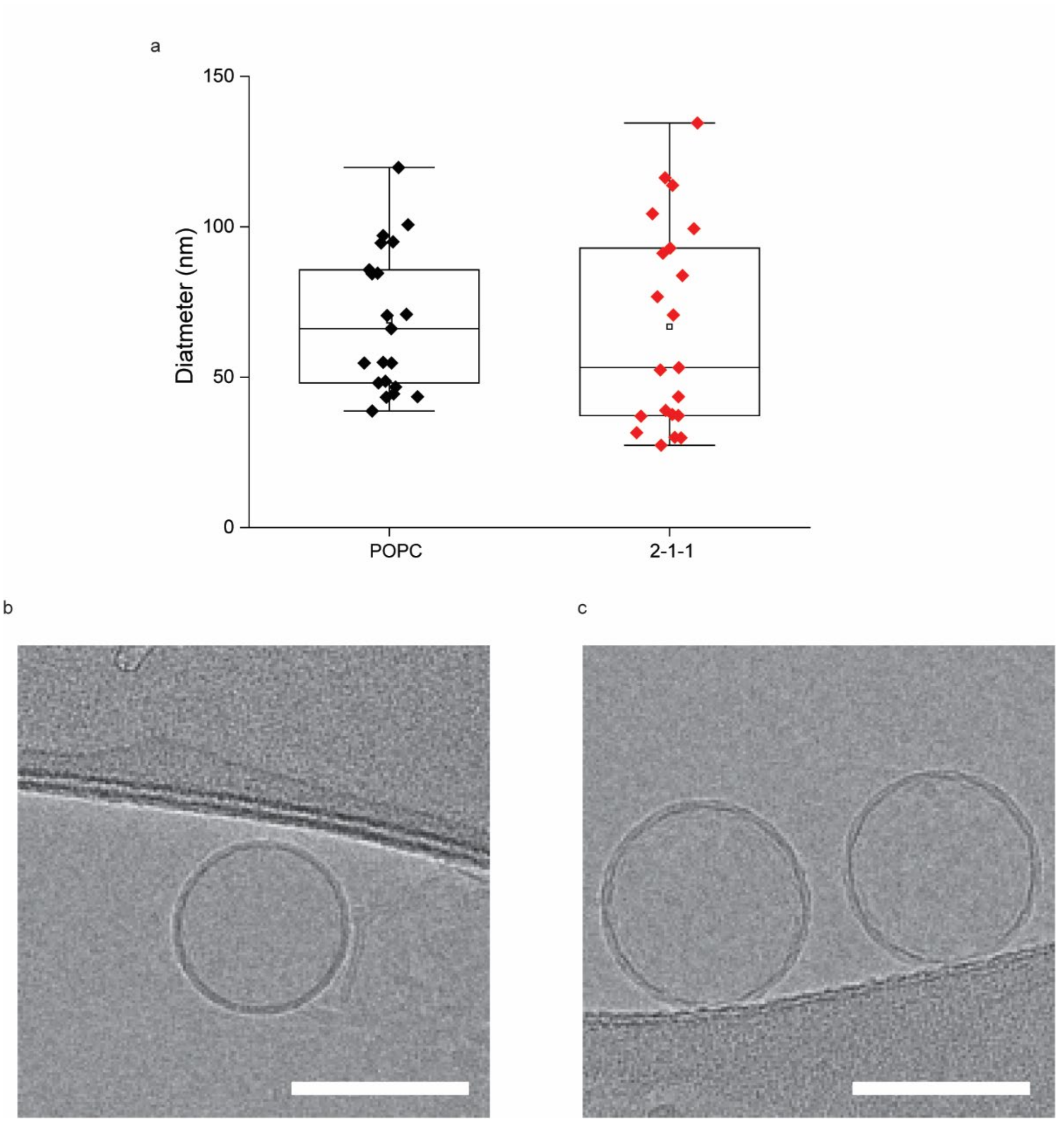

Supplementary Fig. 3 | Diameter of POPC and 2:1:1 liposomes. (a) Box-and-whisker plot (showing mininum, first quartile, median, third quartile and maximum) of liposome diameter from single liposome preparations of POPC and 2:1:1 lipids. The diameters of 22 liposomes were measured from cryo-EM images of each sample. For POPC, the mean diameter is 69 ± 23 nm (±SD), and for 2:1:1, the mean diameter is 67 ± 34 nm (±SD). Source data are provided as a source data file. **(b)** Representative cryo-EM image of a liposome from a single POPC liposome sample prepared using the same method as for the stopped-flow assay. All intact liposomes were measured. White line shows scale for 100 nm. **(c)** Representative cryo-EM image of liposomes

from a single 2:1:1 liposome sample prepared using the same method as for the stopped-flow assay. The first randomly selected 22 intact liposomes were measured.

Supplementary Fig. 4

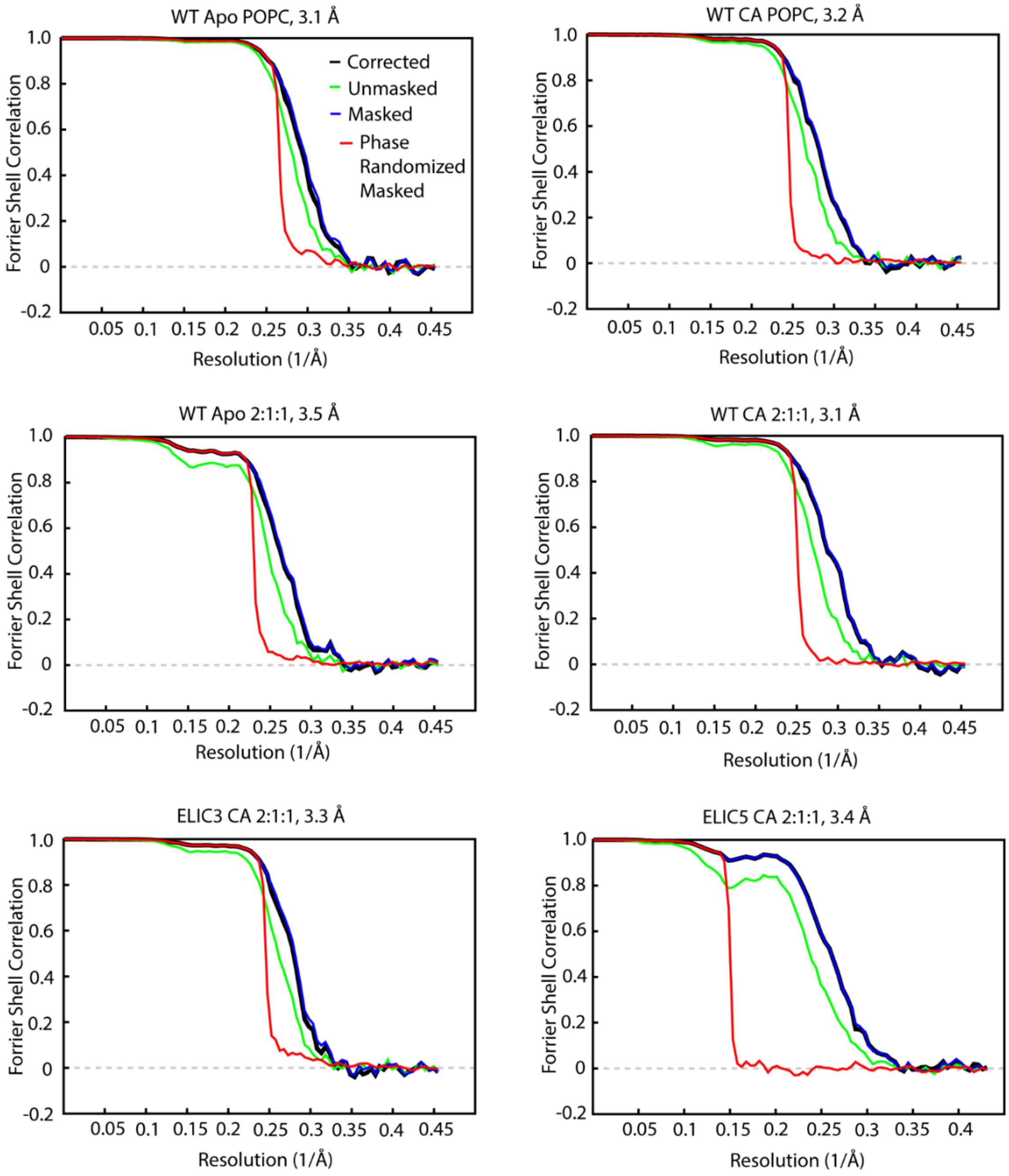

Supplementary Fig. 4 | Fourier shell correlation curves. FSC curves for each ELIC structure labeled according to WT ELIC, ELIC3, ELIC5; apo or with agonist (10 mM cysteamine, CA); the lipid used in the nanodisc (POPC or 2:1:1); and the final post-processed resolution.

Supplementary Fig. 5

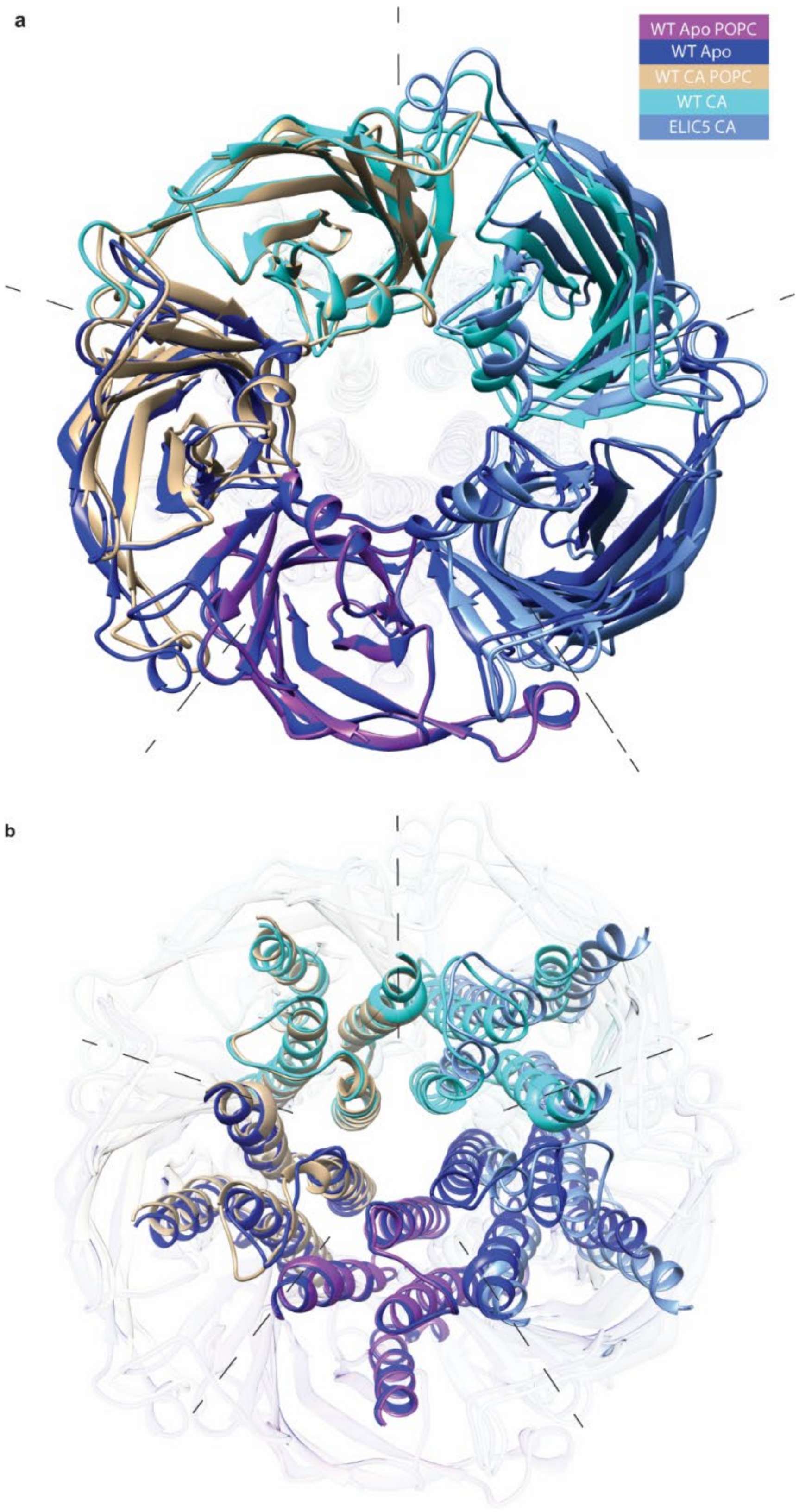

Supplementary Fig. 5 | ELIC structural comparisons (a) Global superposition of ELIC structures showing the ECD of indicated pairs of structures. These include apo POPC (WT apo in POPC nanodiscs, WT apo (WT apo in 2:1:1 nanodiscs), WT CA POPC (WT + 10 mM cysteamine in POPC nanodiscs), WT CA (WT + 10 mM cysteamine in 2:1:1 nanodiscs), ELIC5 CA (ELIC5 + 10 mM cysteamine in 2:1:1 nanodiscs) **(b)** Global superposition of ELIC structures showing, for comparison, the TMD of indicated pairs of structures.

Supplementary Fig. 6

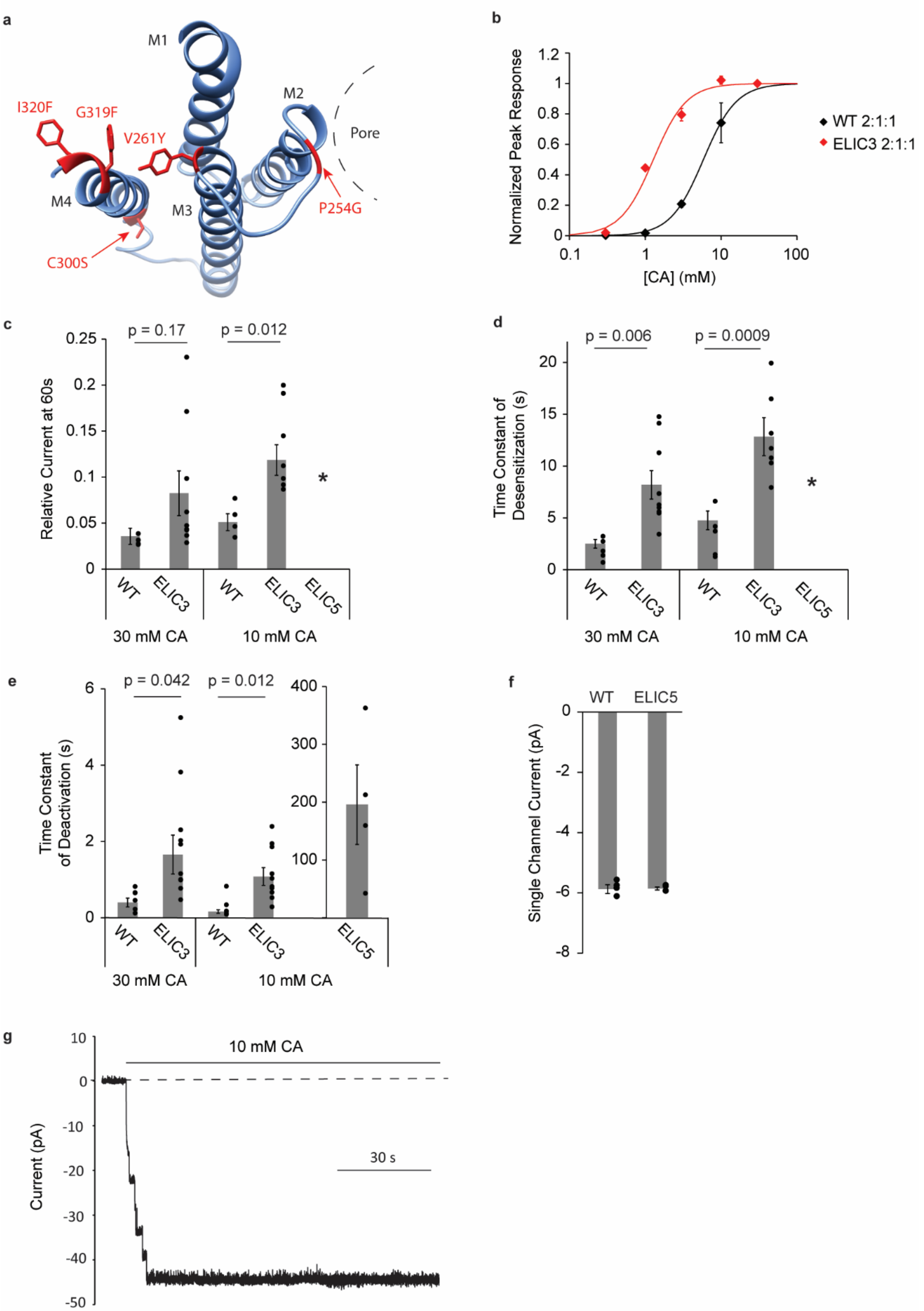

Supplementary Fig. 6 | Functional characterization of ELIC gain-of-function mutants (a) View of one subunit of ELIC TMD from the extracellular side showing the mutated residues (red) for ELIC3 (V261Y, G319F, I320F) and ELIC5 (V261Y, G319F, I320F, P254G and C300S). **(b)** Peak dose response curve of WT ELIC and ELIC3 with cysteamine (CA) obtained from excised patch-clamp recordings from 2:1:1 giant liposomes. Data are fit to a Hill equation with n set to 2, yielding an EC_{50} of 5.2 ± 0.3 mM for WT ELIC and 1.2 ± 0.2 mM for ELIC3 (n = 4). Of note, the recording buffer contained 0.5 mM $BaCl_2$, to maintain patch stability, which leads to ~2-3x right-shift in the EC_{50}[77]. **(c)** Relative current at 60 s (current at 60 s divided by the peak current) after the application of 10 or 30 mM cysteamine from giant liposome recordings of WT ELIC and ELIC3 (n = 4 for both WT conditions, n = 7 and 9 for ELIC3 at 10 and 30 mM CA, respectively). * indicates no data because there was no evidence of desensitization in ELIC5 responses. Statistical significance was determined by an unpaired, two-tailed T-test. **(d)** Same as (c) showing the weighted time constant of desensitization in WT ELIC and ELIC3 currents in response to 10 or 30 mM cysteamine (n = 4 and 5 for WT at 10 and 30 mM CA, respectively, n = 7 and 9 for ELIC3 at 10 and 30 mM CA, respectively). **(e)** Time constant of deactivation after 1 s application of 10 or 30 mM cysteamine in WT ELIC and ELIC3 giant liposome recordings. For ELIC5, 10 mM cysteamine was applied for ~1 min, achieving stable current, prior to removal of agonist (n = 5 for both WT conditions, n = 11 and 10 for ELIC3 at 10 and 30 mM CA, respectively, n = 4 for ELIC5). **(f)** Single channel currents from WT and ELIC5 giant liposome recordings at -60 mV (n = 4). All data are shown as mean ± s.e. for (n) independent experiments. Source data are provided as a source data file. **(g)** Representative ELIC5 response to 10 mM cysteamine from a giant liposome recording, in which single channel openings were resolved. No closing events were observed for ~2 min.

Supplementary Fig. 7

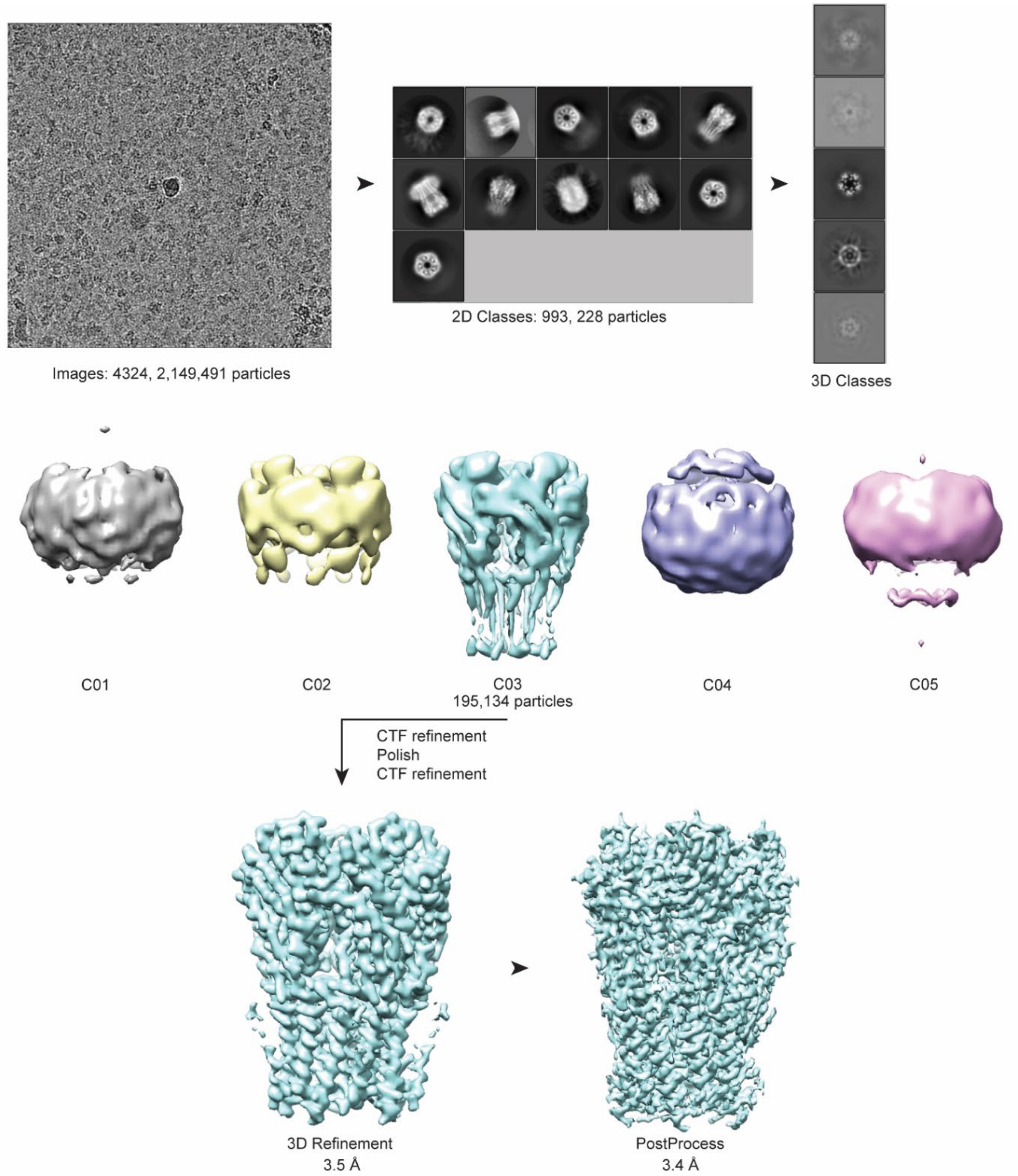

Supplementary Fig. 7 | Diagram for single particle analysis of the ELIC5 CA structure using Relion3. Workflow for analysis showing a representative micrograph, 2D classes, 3D classes and refined and post-processed maps. This workflow for the ELIC5 CA structure is representative of the process for all other structures.

Supplementary Fig. 8

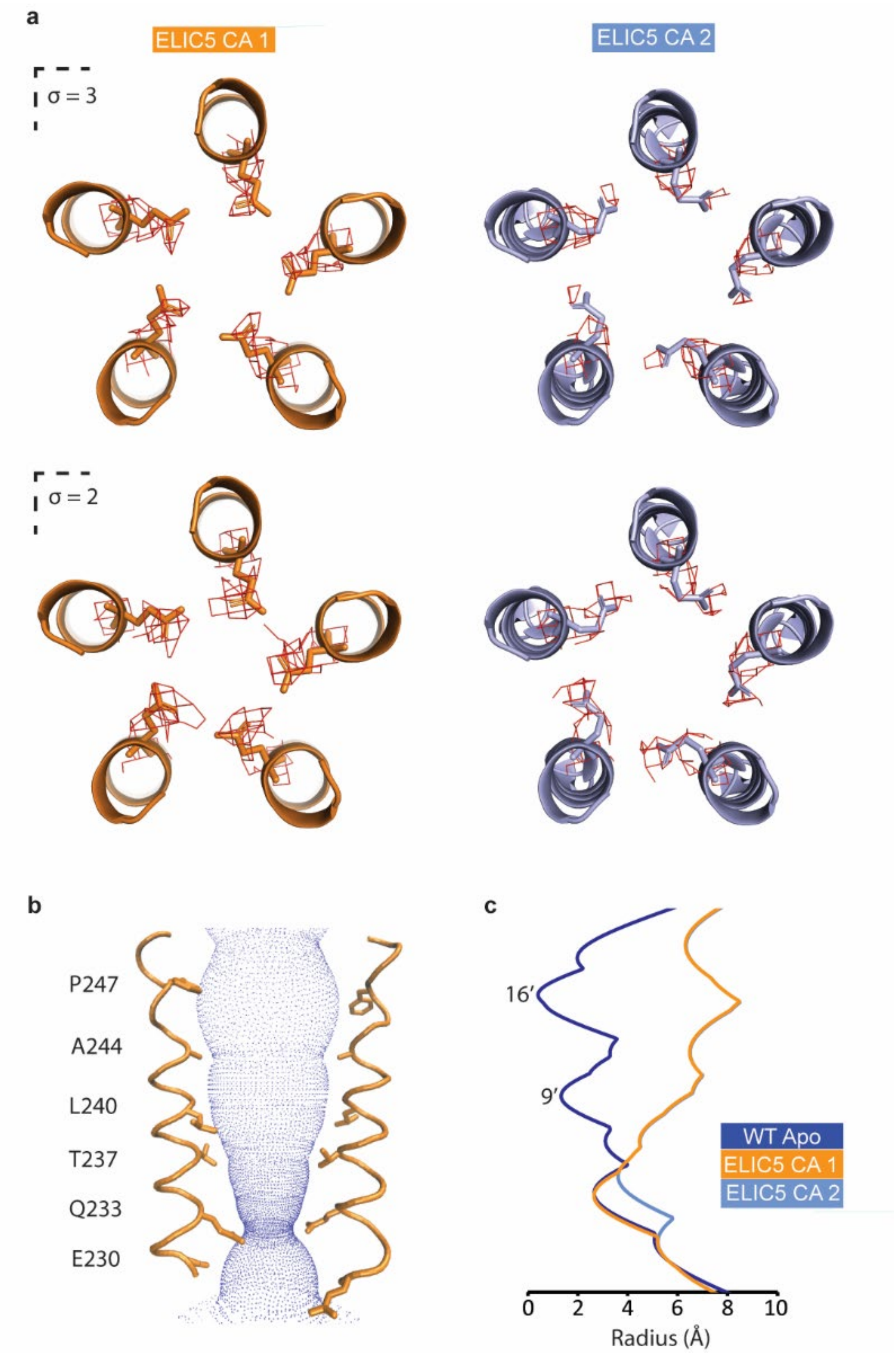

Supplementary Fig. 8 | Two rotamers of Q233 in the ELIC5 CA structure (a) The Q233 side chain shows density for two rotameric states, which were modeled as rotamer 1 (ELIC5 CA 1)

and rotamer 2 (ELIC5 CA 2). Shown are the model and cryo-EM density of the Q233 side chain for ELIC5 CA 1 and ELIC5 CA 2 at σ levels of 3 and 2. Rotamer 1 has a stronger density and was assigned 70% occupancy and rotamer 2, 30% occupancy. **(b)** The ion permeation pathway of ELIC5 CA 1 with pore-facing residues labeled including Q233. ELIC5 CA 2 is shown in Fig. 2e. **(c)** Pore radius as a function of distance along the pore axis of WT Apo and ELIC5 CA with the indicated rotamers. In the ELIC5 CA structure, Q233 forms the narrowest point of the pore with a diameter of 5.6 Å for rotamer 1 and 7.3 Å for rotamer 2.

Supplementary Fig. 9

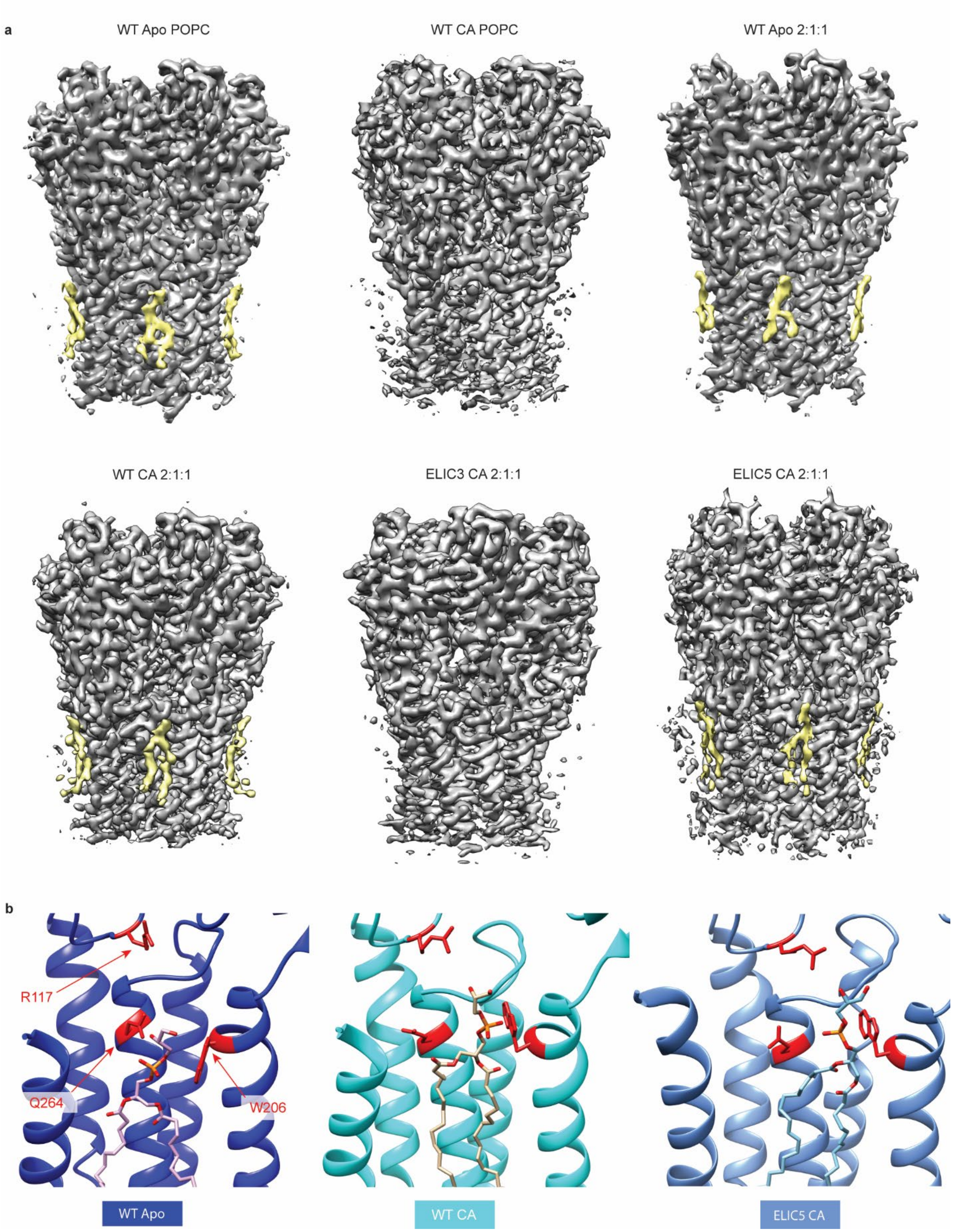

Supplementary Fig. 9 | Bound phospholipids in ELIC structures (a) Cryo-EM density maps of all ELIC structures with and without agonist (10 mM cysteamine) in POPC or 2:1:1 nanodiscs. Shown in yellow is the density for a bound phospholipid illustrating its location at the outer leaflet site. The threshold of the density was adjusted to optimize the appearance of the phospholipid density, and thus the strength of the phospholipid density is not comparable between structures. No significant phospholipid-like densities were detected at other sites. **(b)** Binding modes of the phospholipid in the indicated structures highlighting the position of R117, Q264 and W206 relative to the phospholipid in each structure. Q264 and W206 closely interact with the phospholipid headgroup in the WT apo structure and rotate away from the lipid-protein interface in the WT CA and ELIC5 CA structures. R117 is closest to the phospholipid headgroup in the ELIC5 CA structure.

Supplementary Fig. 10

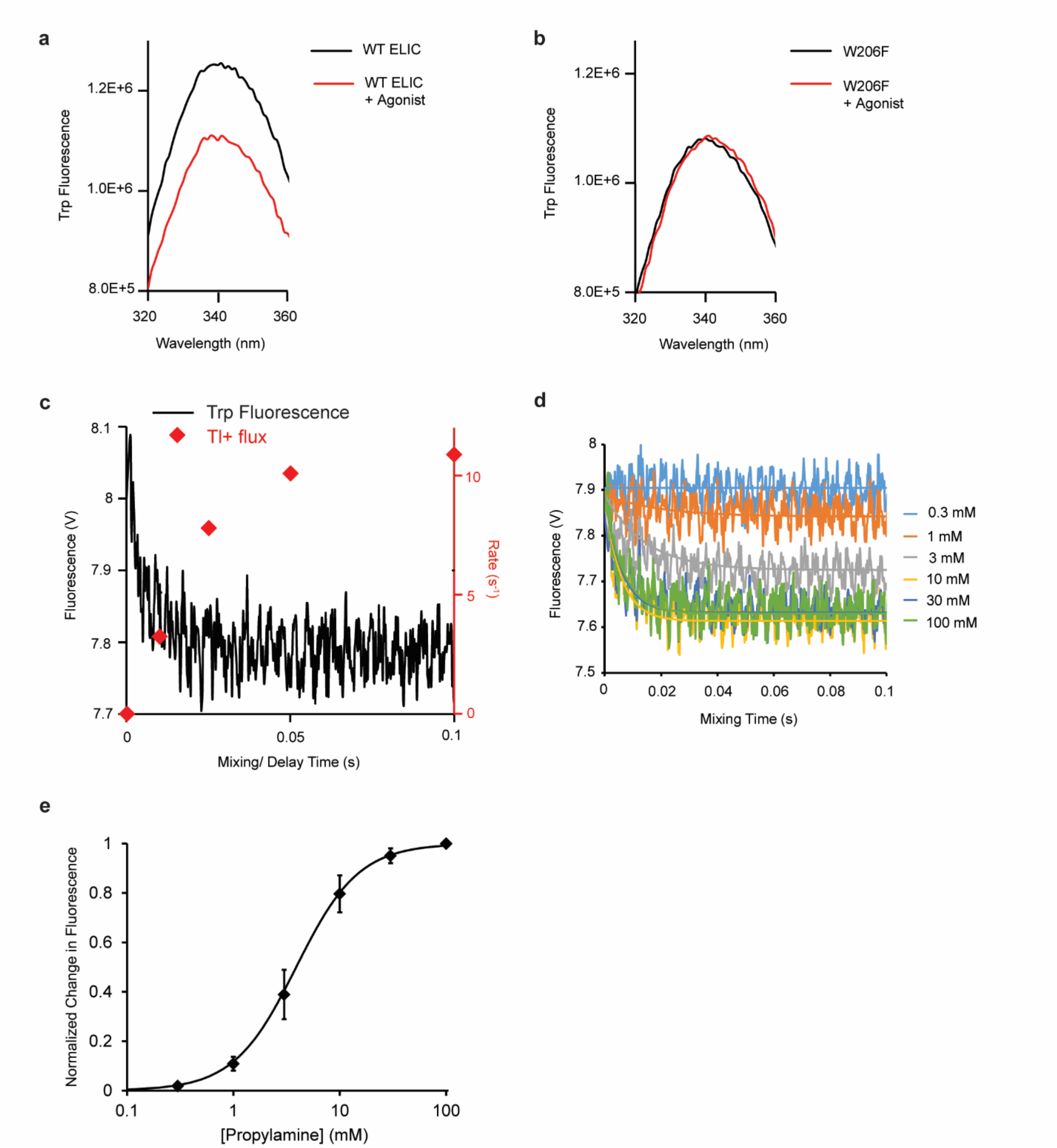

Supplementary Fig. 10 | Stopped-flow tryptophan (TRP) fluorescence measurements of ELIC (a) Emission spectrum of WT ELIC with and without 10 mM propylamine (excitation 295 nm). **(b)** Same as (a) with W206F ELIC. **(c)** Time course of TRP fluorescence of WT ELIC after rapid mixing with 10 mM propylamine (black, time constant = 9.9 ± 0.6 ms, n = 3). The sample

was excited at 295 nm to monitor TRP fluorescence. Also shown is the time course of WT ELIC activation by Tl^+ flux in response to 10 mM propylamine in 2:1:1 liposomes (red, data obtained from Fig. 1, time constant = 41 ± 34 ms, n = 3). In POPC liposomes, the time constant for WT ELIC activation by Tl^+ flux in response to 10 mM propylamine was 20 ± 6 ms (n = 3). **(d)** Representative time courses of TRP fluorescence of WT ELIC after rapid mixing with varying concentrations of propylamine. **(e)** Normalized change in TRP fluorescence derived from time courses as shown in (d). Data are fit to a Hill equation yielding EC_{50} = 4.3 ± 0.8 mM and n = 1.6 ± 0.3 (n = 3). Data are shown as mean ± s.e. for (n) independent experiments. Source data are provided as a source data file. These data indicate that movement of W206 shows a similar dependence on propylamine concentration as channel opening (Supplementary Fig. 1).

Supplementary Fig. 11

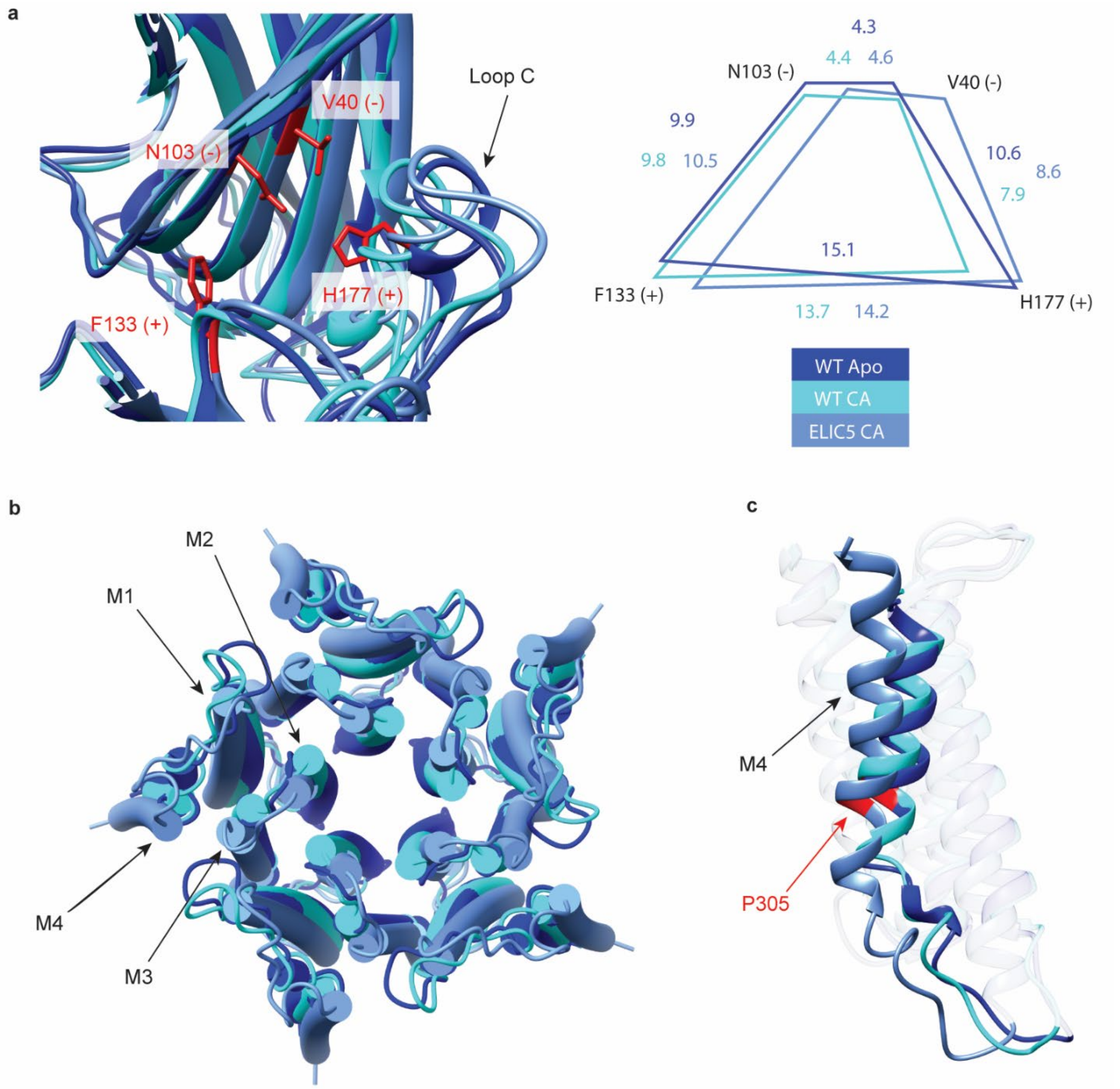

Supplementary Fig. 11 | Comparison of the agonist binding site and TMD between ELIC structures (a) *Left:* Representation of the agonist binding site in WT apo, WT CA and ELIC5 CA structures (global superposition) highlighting the conformational change in loop C. Shown in red are four key residues that form the agonist binding site. *Right*: Distances (Å) between four key residues within the agonist binding site showing contraction of the site in the agonist-bound structures compared to WT apo, and outward translation of the site in the ELIC5 CA structure

compared to WT CA. **(b)** View of the TMD from the intracellular side of WT apo, WT CA and ELIC5 CA structures. **(c)** Comparison of M4 between the indicated structures from a global superposition. Highlighted in red is P305.

Supplementary Fig. 12

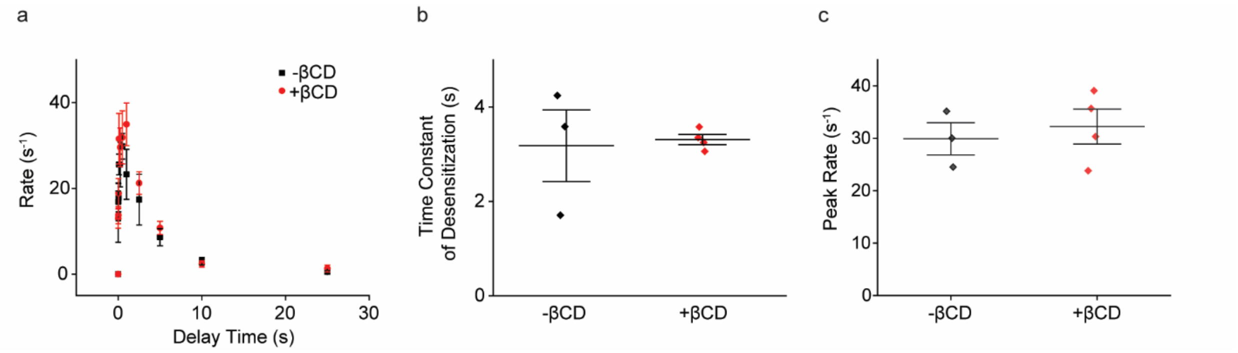

Supplementary Fig. 12 | Effect of methyl-β-cyclodextrin on ELIC responses. (a) Tl^+ flux rates of WT ELIC in 3:1 POPC:POPE liposomes as a function of delay time after mixing with 10 mM propylamine (n=3 for –βCD and n=4 for +βCD). **(b)** Weighted time constant of desensitization in the presence of 10 mM propylamine from Tl^+ flux assay in –βCD and +βCD conditions from (a). **(c)** Peak rate of Tl+ flux in response to 10 mM propylamine in –βCD and +βCD conditions from (a). Data are shown as mean ± s.e. for (n) independent experiments.

Supplementary Fig. 13

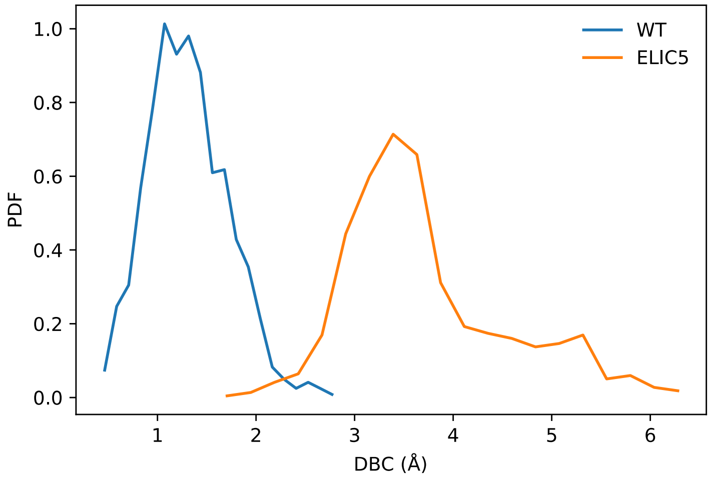

Supplementary Fig. 13 | Distribution of the DBC collective variable from equilibrium MD simulations of POPC bound to WT and ELIC5. The 95th percentile is approximately 6 Å for ELIC5.

Supplementary Fig. 14

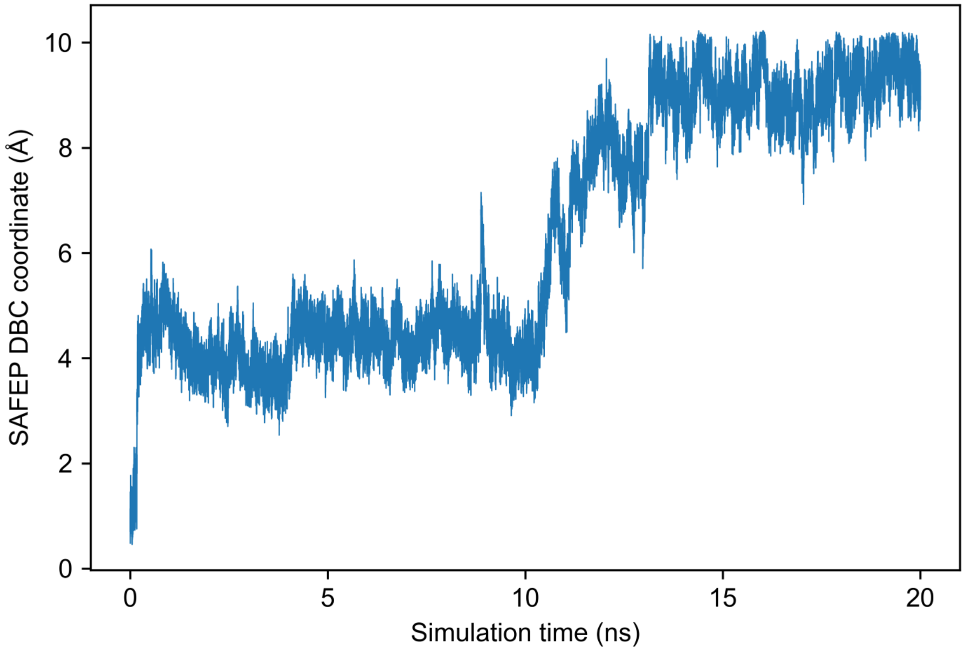

Supplementary Fig. 14 | POPG headgroup distance from bound conformation in the WT apo structure. Equilibrium MD simulation demonstrating spontaneous unbinding of the POPG headgroup from the binding mode of the lipid density in the WT apo structure embedded in a 2:1:1 POPC:POPE:POPG membrane, as measured by SAFEP DBC coordinate, which measures the RMSD of selected headgroup glycerol atoms from an initial bound conformation. No bias is applied to POPG until the SAFEP DBC coordinate reaches 10 Å.

Supplementary Fig. 15

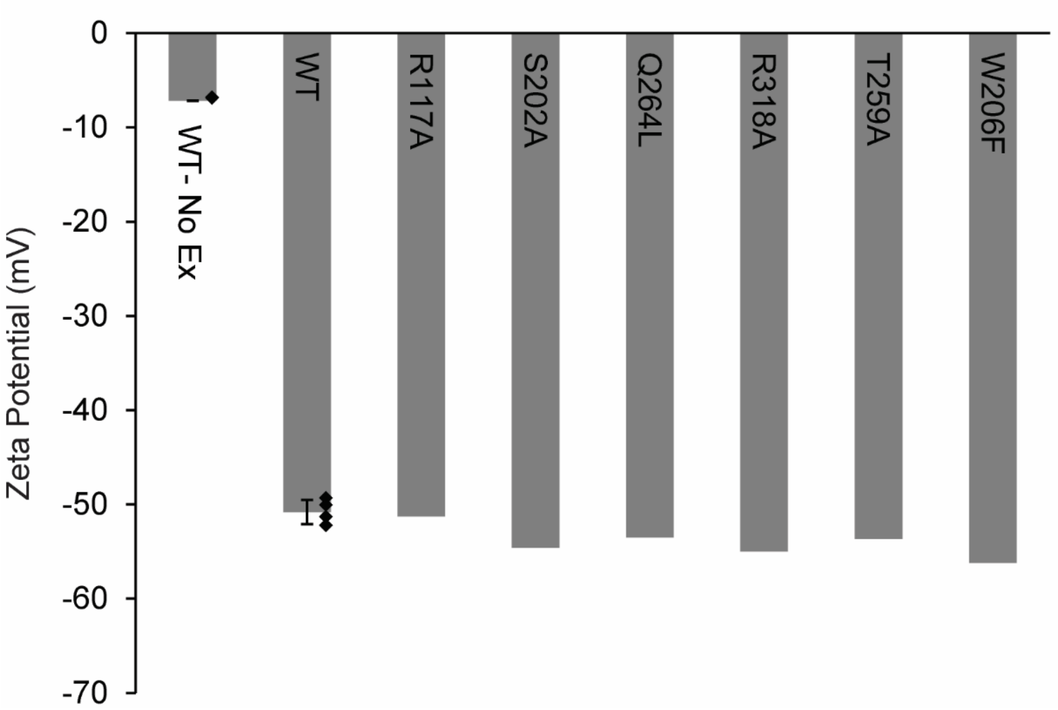

Supplementary Fig. 15 | Zeta potential measurements of mutants. Zeta potential values of WT ELIC in 3:1 POPC:POPE liposome no exchange (“No Ex”) samples (n = 2), WT ELIC (n = 4, same data as Fig. 4b) and the indicated mutants in 3:1 POPC:POPE liposome exchanged (“Ex”) samples (i.e. treated with POPG and methyl-β-cyclodextrin to introduce 25% POPG to the outer leaflet) (n = 1 for each mutant). To verify that POPG exchange was complete in the mutant proteoliposome samples, the zeta potential of one liposome sample for each mutant used in the stopped-flow experiments was checked. Testing a single sample for the mutants was deemed sufficient since the POPG/methyl-β-cyclodextrin treatment consistently produced a zeta potential at least as negative as the WT samples in all six mutant samples, confirming that the exchange procedure is robust and reproducible. For the WT “No Ex” and “Ex” samples, data are shown as mean ± s.e. for (n) independent experiments. Source data are provided as a source data file.

Supplementary Fig. 16

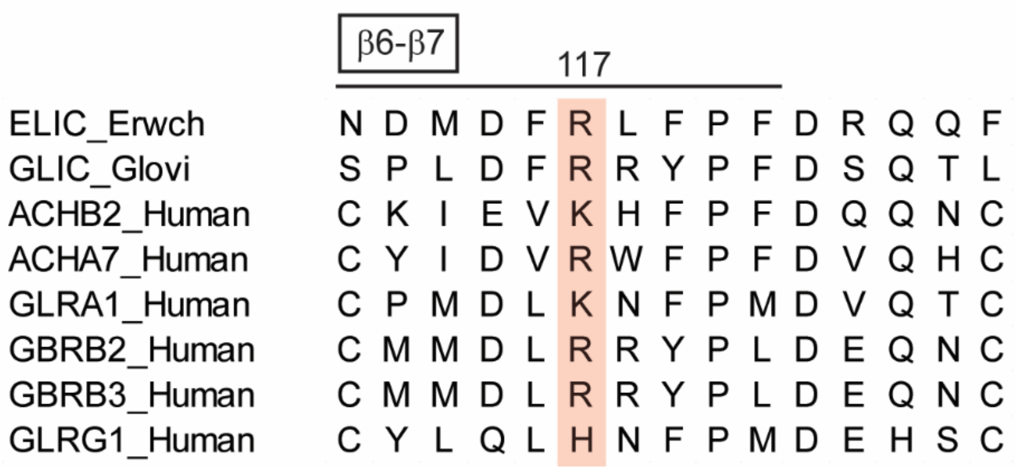

Supplementary Fig. 16 | Sequence alignment comparing the β6-β7 loop in ELIC with GLIC and other human pLGICs. Highlighted is R117 in ELIC.

Supplementary Fig. 17

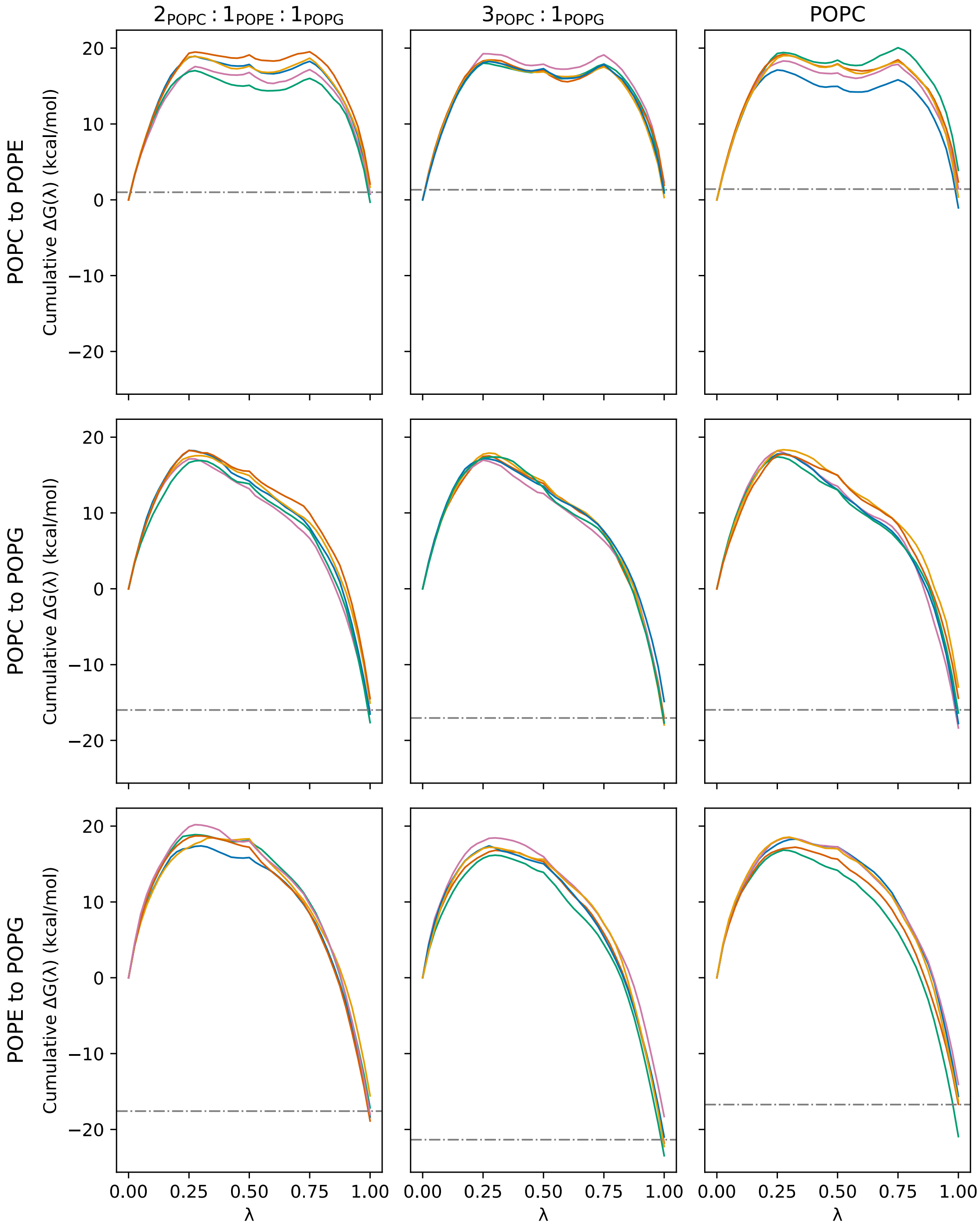

Supplementary Fig. 17 | Cumulative change in free energy vs λ during SAFEP calculation of ΔG_{bulk}. Calculations are shown for at least 5 replicas of three transformations (rows) in three

membrane compositions (columns). Solid lines indicate the cumulative change in free energy with respect to λ for each system, colored by replica. Dashed lines indicate the final average of ΔG_{bulk} values across replicas.

Supplementary Fig. 18

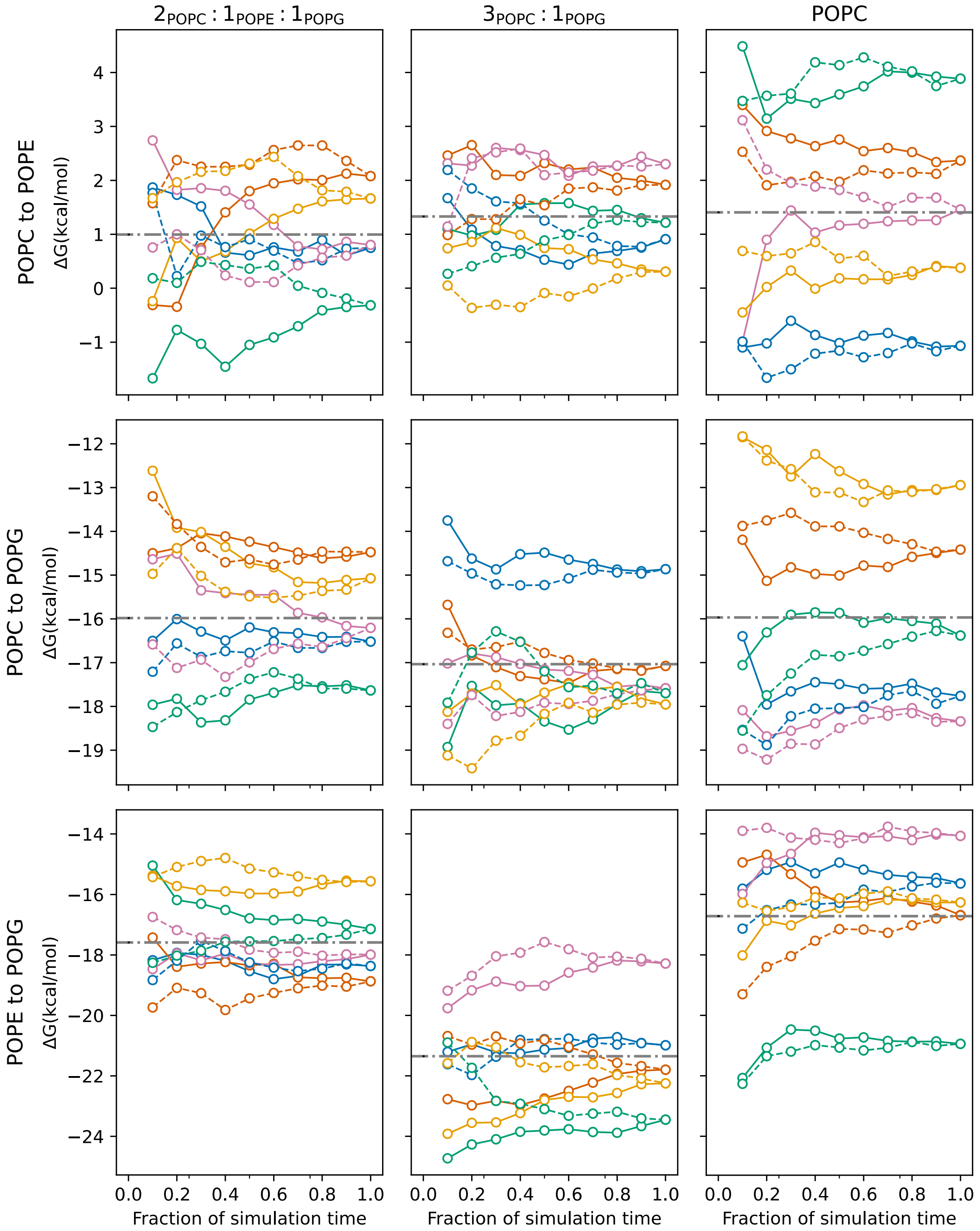

Supplementary Fig. 18 | Dependence of ΔG_{bulk} on the fraction of simulation trajectory used for analysis. Dashed lines indicate reverse-time subsampling (starting from the end of the simulation) while solid lines indicate forward-time subsampling (starting from the end of equilibration for all

windows). Subsamples begin to overlap when the fraction of simulation time reaches 0.5. Colors indicate individual replicas. Dot-dashed lines indicate final average values across replicas.

Supplementary Fig. 19

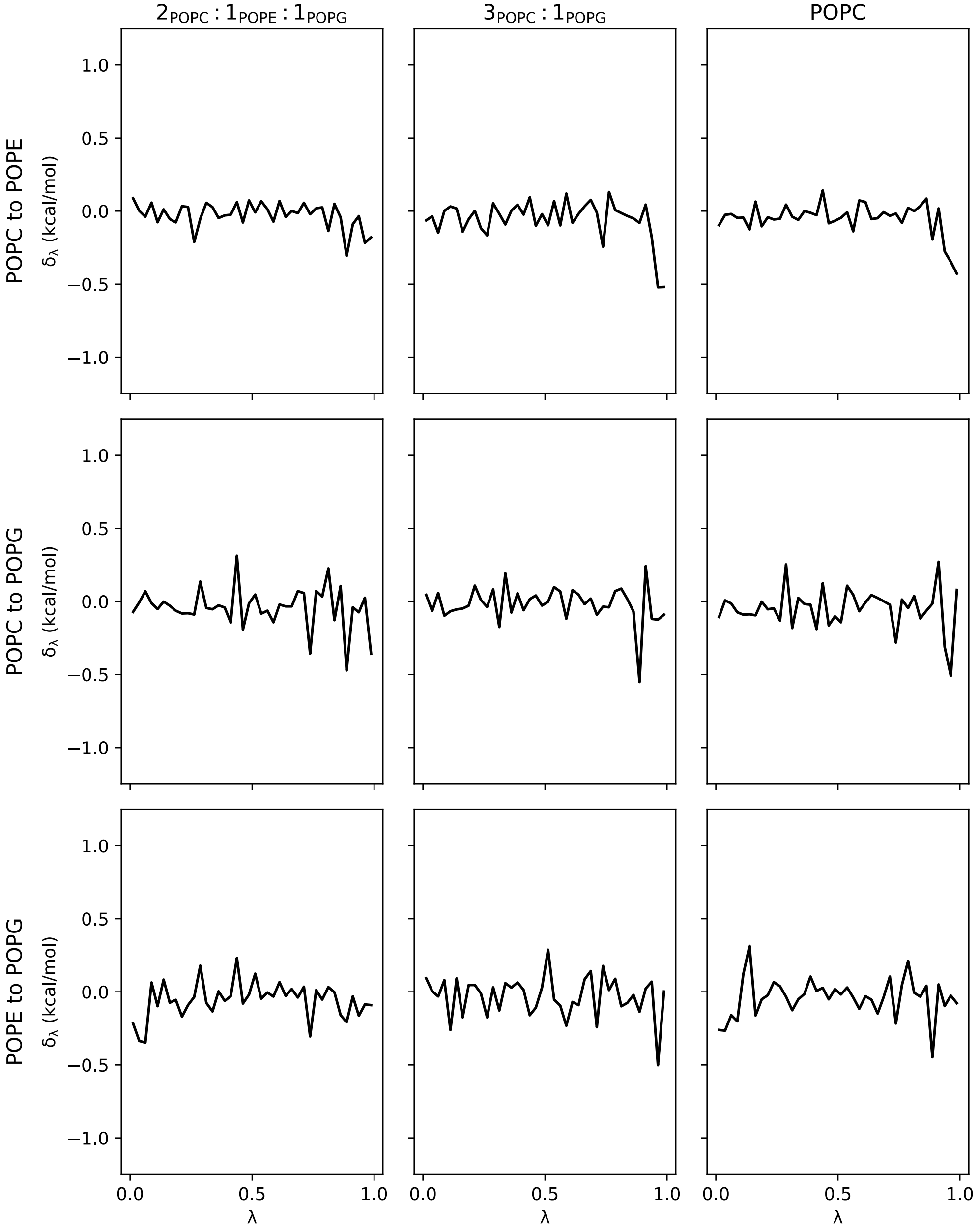

Supplementary Fig. 19 | Hysteresis in the calculation of ΔG_{bulk} as a function of λ. Lines indicate the difference (δ_λ) between the forward and IDWS-generated backward estimates for each window, using an exponential estimator. Colors represent individual replicas; black curves are determined using all replicas. δ_λ values appear to be independent of λ, suggesting the systems are well-equilibrated with respect to λ.

Supplementary Fig. 20

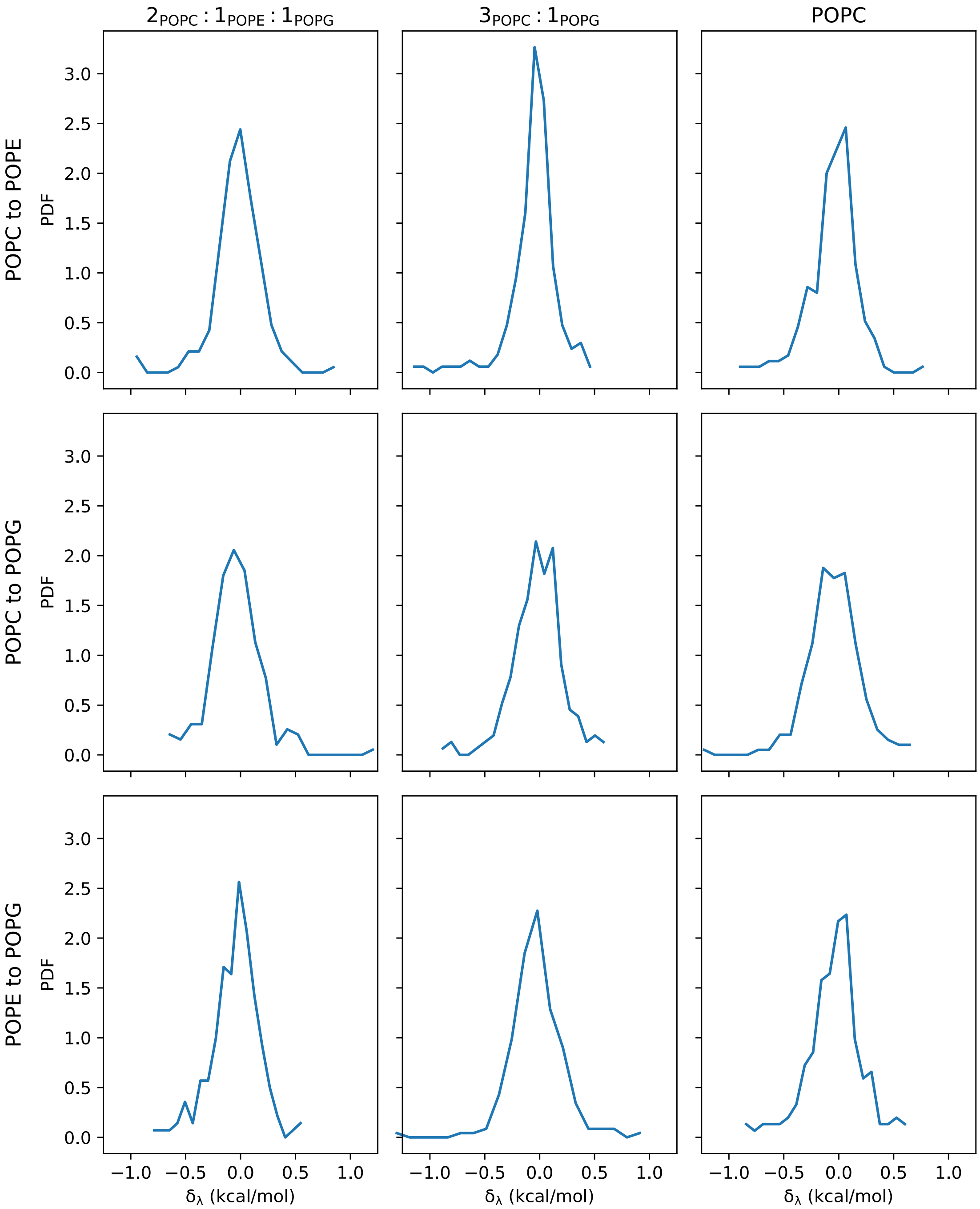

Supplementary Fig. 20 | Distributions of aggregate δ_λ values shown in Supplementary Figure 19. Values are roughly centered around 0, indicating that the aggregate calculations are well-sampled.

Supplementary Figure 21

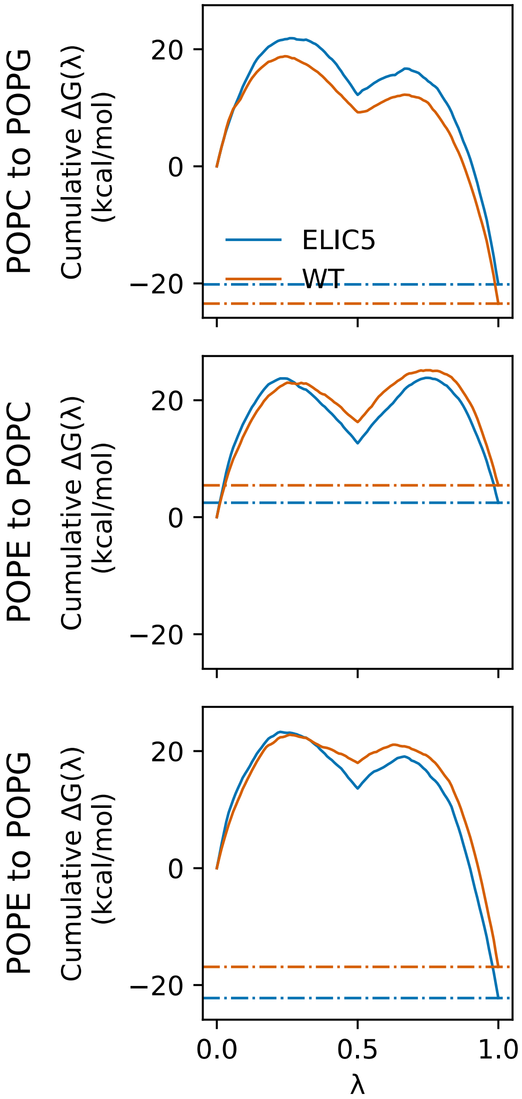

Supplementary Figure 21 | Cumulative change in free energy vs λ during SAFEP calculation of ΔG_M. Calculations are shown for three lipid transformations (rows). Solid lines indicate the cumulative change in free energy with respect to λ for each system, colored by protein structure. Dashed lines indicate the final average of ΔG_M values across replicas, with one exception. As expected, all paths are reasonably smooth; a strong cusp at 0.5 is not unusual because that is the inflection point for both electrostatics and Van der Waals interactions in the protein FEP calculations.

Supplementary Figure 22

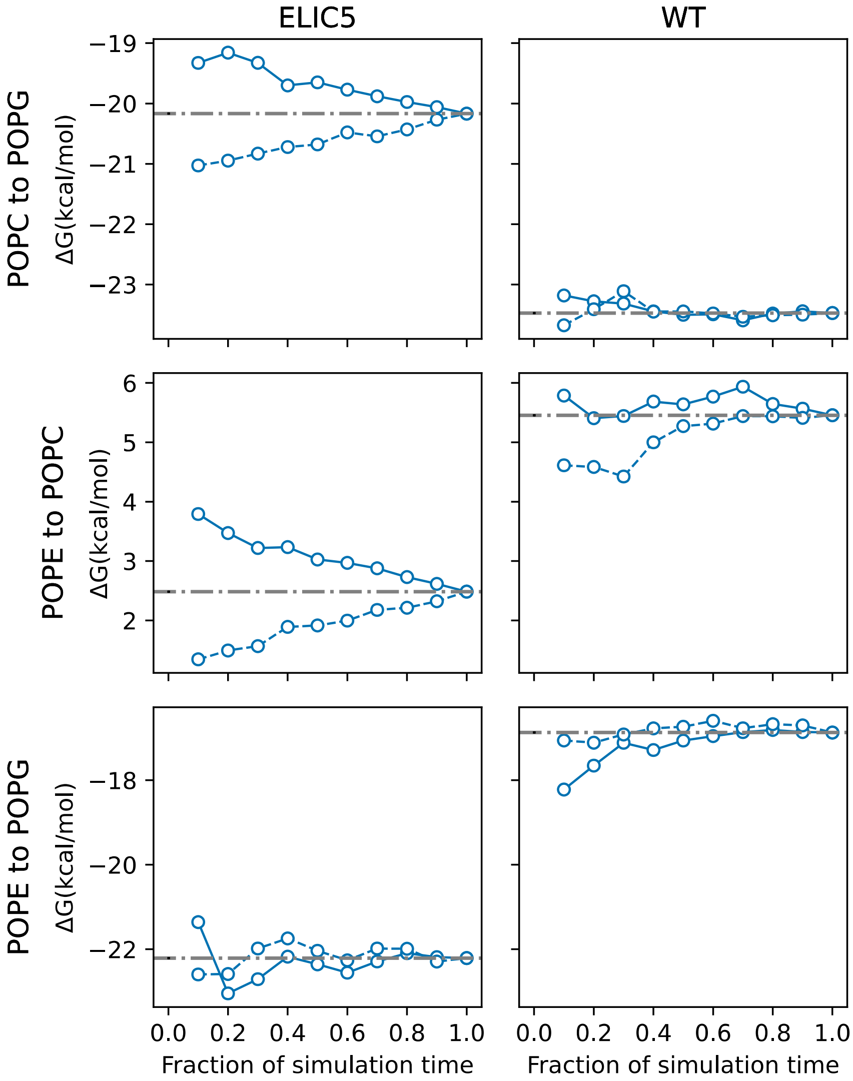

Supplementary Figure 22 | Dependence of ΔG_M on the fraction of simulation trajectory used for analysis, for three lipid transformations (rows) and two protein conformations (columns). Dashed lines indicate reverse-time subsampling (starting from the end of the simulation) while solid lines indicate forward-time subsampling (starting from the end of equilibration for all windows). Subsamples begin to overlap when the fraction of simulation time reaches 0.5. Colors indicate individual replicas. Dot-dashed lines indicate final average values across replicas. Well-converged systems are those with a discrepancy of at most 1 kcal/mol at 50% simulation time.

Supplementary Figure 23

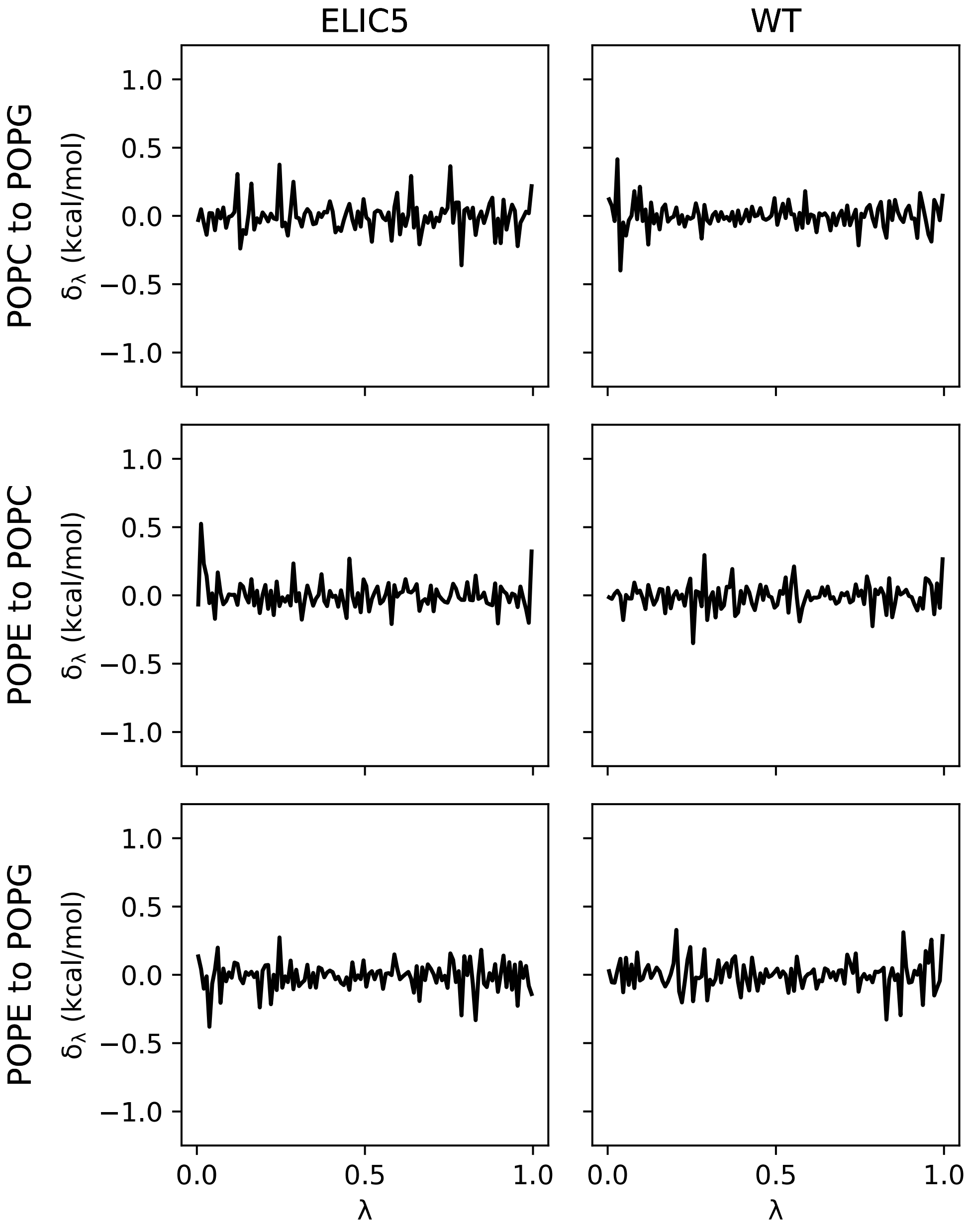

Supplementary Figure 23 | Hysteresis in the calculation of ΔG_M as a function of λ. Lines indicate the difference (δ_λ) between the forward and IDWS-generated backward estimates for each window, using an exponential estimator. δ_λ values appear to be independent of λ, suggesting the systems are well-equilibrated with respect to λ.

Supplementary Figure 24

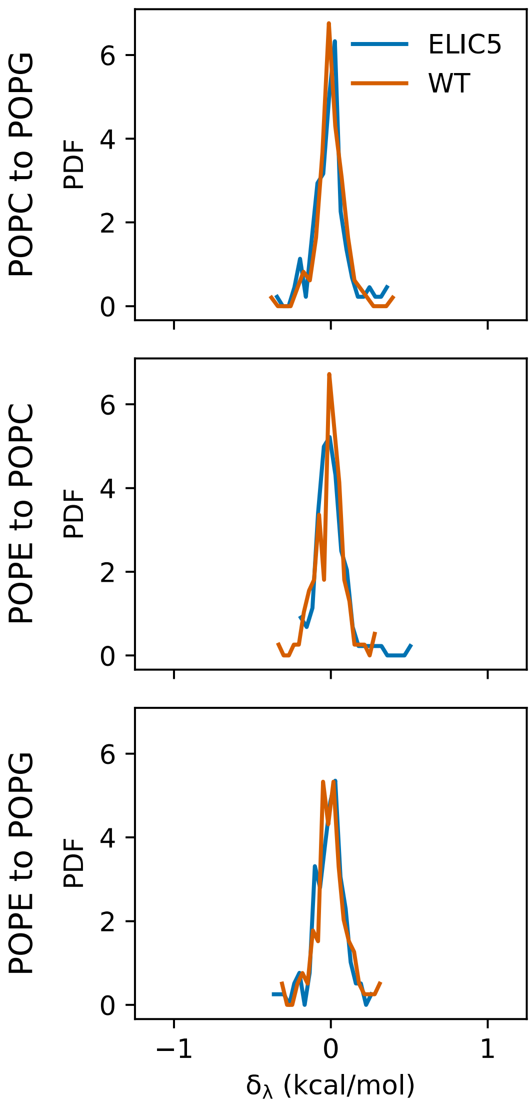

Supplementary Figure 24 | Distributions of aggregate protein δ_λ values shown in Supplementary Figure 23. Lines indicate the approximate probability density function for the distribution of each transformation and system (ELIC5 in blue, WT in orange). All δ_λ distributions are symmetric and strongly peaked around 0. The distributions are much narrower than for the membrane calculations, due to the use of significantly more windows (120 vs. 40).
