# Supplementary material for: Open-channel structure of a pentameric ligand-gated ion channel reveals a mechanism of leaflet-specific phospholipid modulation
Source: Nat Commun. 2022 Nov 17;13:7017. doi: 10.1038/s41467-022-34813-5 (PMC9668969; doi:10.1038/s41467-022-34813-5)
Supplement: Supplementary file 3 — Description of Additional Supplementary Files [file 41467_2022_34813_MOESM3_ESM.pdf]

Description of Additional Supplementary Files

File name: Supplementary Movie 1
Description: Trajectory showing a morph between the WT Apo and ELIC5 CA structures focused on the ECD-TMD interface of two adjacent subunits. The movie starts with WT Apo, morphs to ELIC5 CA, and then back to WT Apo.

File name: Supplementary Movie 2
Description: Trajectory showing a morph between the WT CA and ELIC5 CA structures focused on the ECD-TMD interface of two adjacent subunits. The movie starts with WT CA, morphs to ELIC5 CA, and then back to WT CA.

File name: Supplementary Movie 3
Description: Trajectory showing a morph between the WT Apo and ELIC5 CA structures showing just the TMD down the pore axis of the channel (viewed from the extracellular end). The movie starts with WT Apo, morphs to ELIC5 CA, and then back to WT Apo.

File name: Supplementary Movie 4
Description: Trajectory showing a morph between the WT CA and ELIC5 CA structures showing just the TMD down the pore axis of the channel (viewed from the extracellular end). The movie starts with WT CA, morphs to ELIC5 CA, and then back to WT CA.

File name: Supplementary Movie 5
Description: Trajectory showing a morph between the WT Apo and ELIC5 CA structures with a profile view (side view of the ECD and TMD). The movie starts with WT Apo, morphs to ELIC5 CA, and then back to WT Apo.

File name: Supplementary Movie 6
Description: Trajectory showing a morph between the WT CA and ELIC5 CA structures with a profile view (side view of the ECD and TMD). The movie starts with WT CA, morphs to ELIC5 CA, and then back to WT CA.

File name: Supplementary Movie 7
Description: Trajectory showing a morph between the WT Apo and ELIC5 CA structures viewed from the ECD down the pore axis of the channel. The movie starts with WT Apo, morphs to ELIC5 CA, and then back to WT Apo.

File name: Supplementary Movie 8
Description: Trajectory showing a morph between the WT CA and ELIC5 CA structures viewed from the ECD down the pore axis of the channel. The movie starts with WT CA, morphs to ELIC5 CA, and then back to WT CA.
