# Supplementary material for: Open-channel structure of a pentameric ligand-gated ion channel reveals a mechanism of leaflet-specific phospholipid modulation
Source: Nat Commun. 2022 Nov 17;13:7017. doi: 10.1038/s41467-022-34813-5 (PMC9668969; doi:10.1038/s41467-022-34813-5)
Supplement: Supplementary file 12 — Reporting Summary [file 41467_2022_34813_MOESM12_ESM.pdf]

nature portfolio

Corresponding author(s): Wayland W. L. Cheng

Last updated by author(s): Nov 2, 2022

Reporting Summary

Nature Portfolio wishes to improve the reproducibility of the work that we publish. This form provides structure for consistency and transparency in reporting. For further information on Nature Portfolio policies, see our Editorial Policies and the Editorial Policy Checklist.

Statistics

For all statistical analyses, confirm that the following items are present in the figure legend, table legend, main text, or Methods section.

n/a	Confirmed	
☐	☒	The exact sample size (*n*) for each experimental group/condition, given as a discrete number and unit of measurement
☐	☒	A statement on whether measurements were taken from distinct samples or whether the same sample was measured repeatedly
☐	☒	The statistical test(s) used AND whether they are one- or two-sided<br>*Only common tests should be described solely by name; describe more complex techniques in the Methods section.*
☒	☐	A description of all covariates tested
☐	☒	A description of any assumptions or corrections, such as tests of normality and adjustment for multiple comparisons
☐	☒	A full description of the statistical parameters including central tendency (e.g. means) or other basic estimates (e.g. regression coefficient) AND variation (e.g. standard deviation) or associated estimates of uncertainty (e.g. confidence intervals)
☐	☒	For null hypothesis testing, the test statistic (e.g. *F*, *t*, *r*) with confidence intervals, effect sizes, degrees of freedom and *P* value noted<br>*Give P values as exact values whenever suitable.*
☒	☐	For Bayesian analysis, information on the choice of priors and Markov chain Monte Carlo settings
☒	☐	For hierarchical and complex designs, identification of the appropriate level for tests and full reporting of outcomes
☒	☐	Estimates of effect sizes (e.g. Cohen's *d*, Pearson's *r*), indicating how they were calculated

Our web collection on statistics for biologists contains articles on many of the points above.

Software and code

Policy information about availability of computer code

Data collection	1. Patch-clamp data: pClamp10.4<br>2. Fluorescence stopped-flow data: AppliedPhotophysics Pro-Data SX software<br>3. Cryo-EM image acquisition: EPU 2.9.0.1519 Thermo Fisher Scientific.
Data analysis	1. Electrophysiology data: Clampfit10.4<br>2. Stopped-flow traces: Origin2019b<br>3. CTF estimation: GCTF version 1.06<br>4. Single particle analysis: Relion 3.1<br>5. Pore profile analysis: HOLE 2.0<br>6. 3D volume visualization: UCSF Chimera 1.14<br>7. Generation of molecular images: PyMOL 2.5.2 and UCSF Chimera 1.14<br>8. Model building: COOT 0.9.8.1<br>9. Structure refinement: Phenix 1.19.2-4158<br>10. Motion correction: MotionCor2<br>11. Zeta potentials: Zetasizer nano software v3.30<br>12. MD simulations: NAMD 2.14<br>13. Analysis scripts for FEP: https://github.com/BranniganLab/ELIC-phospholipid-identification. DOI: 10.5281/zenodo.7264978

For manuscripts utilizing custom algorithms or software that are central to the research but not yet described in published literature, software must be made available to editors and reviewers. We strongly encourage code deposition in a community repository (e.g. GitHub). See the Nature Portfolio guidelines for submitting code & software for further information.

Data

Policy information about availability of data

All manuscripts must include a data availability statement. This statement should provide the following information, where applicable:

- Accession codes, unique identifiers, or web links for publicly available datasets
- A description of any restrictions on data availability
- For clinical datasets or third party data, please ensure that the statement adheres to our policy

The data supporting the findings of this study are available within the paper and supplementary information files. The cryo-EM maps have been deposited in the Electron Microscopy Data Bank (EMDB) under accession codes EMD-27216 (WT CA POPC), EMD-27215 (WT Apo POPC), EMD-27218 (WT CA 2:1:1), EMD-27217 (WT Apo 2:1:1), EMD-27219 (ELIC3 CA 2:1:1), EMD-27220 (ELIC5 CA 2:1:1). The structural coordinates have been deposited in the RCSB Protein Data Bank (PDB) under the accession codes 8D64 (WT CA POPC), 8D63 (WT Apo POPC), 8D66 (WT CA 2:1:1), 8D65 (WT Apo 2:1:1), 8D67 (ELIC3 CA 2:1:1), and 8D68 (ELIC5 CA 2:1:1). Initial model building of the ELIC CA 2:1:1 structure used the ELIC crystal structure, PDB 2YN6 ([https://www.wwpdb.org/pdb?id=pdb_00002yn6]). A source data file is also provided for Figures 1, 4, and 6, and Supplementary Figures 1, 2, 3, 6, 10 and 15.

Human research participants

Policy information about studies involving human research participants and Sex and Gender in Research.

Reporting on sex and gender	N/A
Population characteristics	N/A
Recruitment	N/A
Ethics oversight	N/A

Note that full information on the approval of the study protocol must also be provided in the manuscript.

Field-specific reporting

Please select the one below that is the best fit for your research. If you are not sure, read the appropriate sections before making your selection.

☒ Life sciences ☐ Behavioural & social sciences ☐ Ecological, evolutionary & environmental sciences

For a reference copy of the document with all sections, see nature.com/documents/nr-reporting-summary-flat.pdf

Life sciences study design

All studies must disclose on these points even when the disclosure is negative.

Sample size	No statistical method was used to determine sample size. Sample size for the fluorescence stopped-flow experiments, electrophysiology recordings, and zeta potential measurements were considered sufficient based on prior studies that used similar methodology. For the mutant "exchanged" samples in Supplementary Fig. 15, zeta potentials were measured for the first liposome sample used for the fluorescence stopped-flow studies. This was deemed sufficient since all six mutant proteoliposome samples produced a zeta potential as negative as the WT "exchanged" samples, demonstrating that the exchange procedure is robust and reproducible. For the liposome size measurements by cryo-EM, this was performed with a single liposome preparation using either POPC or 2:1:1 lipid. This was deemed sufficient since the size distribution of liposomes was consistent with prior reports as measured by cryo-EM and the measurements showed no gross difference in liposome size between the two lipid conditions.
Data exclusions	No data was excluded from analysis.
Replication	Fluorescence stopped-flow experiments were performed in triplicate or more independent replicates as indicated in the figure legend with each experiment being a new proteoliposome sample. The replicates from the electrophysiology experiment are from separate patch-clamp recordings taken from a single giant liposome preparation (number of recordings is indicated in the figure legend). The tryptophan stopped-flow data are from three independent replicates. The zeta potential measurements were obtained from liposome samples prepared independently. All attempts at replication were successful. Structure determination was not performed in replicates, which is standard practice.
Randomization	Randomization is not applicable to the structural studies. For the electrophysiology experiments, different liposomes are randomly selected for each recording. For the fluorescence stopped-flow experiments, there is no variable in the sample preparation that should be subject to randomization.
Blinding	Acquisition and analysis of functional data were objectively performed in the same way for all conditions. Blinding was not deemed necessary based on prior studies that used similar methodology.

Reporting for specific materials, systems and methods

We require information from authors about some types of materials, experimental systems and methods used in many studies. Here, indicate whether each material, system or method listed is relevant to your study. If you are not sure if a list item applies to your research, read the appropriate section before selecting a response.

Materials & experimental systems

n/a	Involved in the study
☒	☐ Antibodies
☒	☐ Eukaryotic cell lines
☒	☐ Palaeontology and archaeology
☒	☐ Animals and other organisms
☒	☐ Clinical data
☒	☐ Dual use research of concern

Methods

n/a	Involved in the study
☒	☐ ChIP-seq
☒	☐ Flow cytometry
☒	☐ MRI-based neuroimaging
